# Cannabinol’s Modulation of Genes Involved in Oxidative Stress Response and Neuronal Plasticity: A Transcriptomic Analysis

**DOI:** 10.3390/antiox14060744

**Published:** 2025-06-17

**Authors:** Serena Silvestro, Marco Calabrò, Alessandra Trainito, Stefano Salamone, Federica Pollastro, Emanuela Mazzon, Aurelio Minuti

**Affiliations:** 1 IRCCS Centro Neurolesi “Bonino-Pulejo”, Via Provinciale Palermo, Contrada Casazza, 98124 Messina, Italy; serena.silvestro@irccsme.it (S.S.); marco.calabro@irccsme.it (M.C.); alessandra.trainito92@gmail.com (A.T.); aurelio.minuti@irccsme.it (A.M.); 2 Department of Pharmaceutical Sciences, University of Eastern Piedmont, Largo Donegani 2, 28100 Novara, Italy; salamone.ste@gmail.com (S.S.); federica.pollastro@uniupo.it (F.P.); 3 Department of Medical, Oral and Biotechnological Sciences, University “G. d’Annunzio” Chieti-Pescara, 66100 Chieti, Italy

**Keywords:** Cannabinol, transcriptomics, NSC-34, oxidative stress, neuronal plasticity

## Abstract

*Cannabis sativa* is a remarkable source of bioactive compounds, with over 150 distinct phytocannabinoids identified to date. Among these, cannabinoids are gaining attention as potential therapeutic agents for neurodegenerative diseases. Previous research showed that cannabinol (CBN), a minor cannabinoid derived from Δ^9^-tetrahydrocannabinol, exhibits antioxidant, anti-inflammatory, analgesic, and anti-bacterial effects. The objective of this study was to assess the protective potential of 24 h CBN pre-treatment, applied at different concentrations (5 µM, 10 µM, 20 µM, 50 µM, and 100 µM), in differentiated neuroblastoma × spinal cord (NSC-34) cells. Transcriptomic analysis was performed using next-generation sequencing techniques. Our results reveal that CBN had no negative impact on cell viability at the tested concentrations. Instead, it showed a significant effect on stress response and neuroplasticity-related processes. Specifically, based on the Reactome database, the biological pathways mainly perturbed by CBN pre-treatment were investigated. This analysis highlighted a significant enrichment in the Reactome pathway’s cellular response to stress, cellular response to stimuli, and axon guidance. Overall, our results suggest that CBN holds promise as an adjuvant agent for neurodegenerative diseases by modulating genes involved in neuronal cell survival and axon guidance.

## 1. Introduction

*Cannabis sativa* is a plant with multifaceted, recognized applications. It has a rich history, spanning more than 4000 years, of being utilized for religious, recreational, and therapeutic purposes [1]. In recent decades, the discovery of cannabis’s chemical composition and the ability to isolate its pure compounds sparked great interest within the scientific community: the plant contains over 500 distinct compounds, with around 125 of them identified and classified as phytocannabinoids [1]. Numerous studies, both preclinical and clinical, suggested that phytocannabinoids could be effective in treating a broad range of conditions, including neurological conditions, pain, cancer, and psychiatric disorders [2,3,4]. The pharmacological actions of phytocannabinoids are primarily mediated through their interaction with the endocannabinoid system (ECS), a signaling system used by the central and peripheral nervous system [5,6]. ECS consists of cannabinoid receptors, endocannabinoids, and the enzymes responsible for their production and breakdown [7,8]. There are two primary types of cannabinoid receptors, type I (CNR1) and type II (CNR2), both of which are part of the seven transmembrane domain, G-protein-coupled receptor superfamily. CNR1 is seemingly involved in regulating pain, emotions, motor control, memory, nausea, appetite, vomiting, as well as reward and pleasure [6]. CNR2 was initially observed only under pathological conditions, such as Alzheimer’s disease [9], multiple sclerosis, and amyotrophic lateral sclerosis [10], possibly suggesting an association with these diseases. However, later investigations have also detected these receptors in neurons under physiological conditions [11].

These observations support the critical value of ECS signaling pathways and promote the research and development of drugs targeting this system [12]. Among the investigated compounds, a particular focus has been given to both major and minor phytocannabinoids [13]. Indeed, these compounds are now being suggested as neuroprotective agents. Particularly, phytocannabinoids exert their effects by reducing oxidative stress, enhancing the scavenging of a broad spectrum of free radicals, and inhibiting apoptosis. Oxidative stress arises from an overproduction of reactive oxygen (ROS) and nitrogen species (RNS), which can harm cellular components, especially mitochondria, ultimately triggering the buildup of toxic molecules, cell death, and inflammation [14]. The brain is especially vulnerable to the ROS effects because of its high lipid content, intense metabolic demand, limited regenerative ability, and relatively low antioxidant defenses [14]. Several studies have shown that oxidative stress and inflammation are correlated with neurodegenerative diseases [15]. For instance, individuals with mild cognitive impairment (MCI) have been found to exhibit notably higher levels of oxidative stress markers in the peripheral system compared to healthy individuals [16], and oxidative stress has been recognized as a contributor to faster cognitive decline with aging [17,18].

In recent years, cannabinoids have been found to influence cell survival by either promoting or inhibiting cell death [19]. Notably, they have also been shown to promote neurogenesis [20,21] and to exert critical effects on developmental processes such as axon guidance [22].

The literature to date predominantly focused on major phytocannabinoids. However, minor compounds, such as cannabinol (CBN), also have the potential to interact with ECS [13]. Therefore, this study investigated whether CBN can exert effects comparable to those of its major counterparts. CBN is produced through the oxidative degradation of Δ^9^-THC, which occurs upon exposure to light, oxygen, and heat. This reaction causes the Δ^9^-double bond to shift, resulting in the formation of a fully aromatic compound [23]. Notably, previous experimental studies demonstrated that CBN has antioxidant [24], analgesic [25], anti-inflammatory [26], and anti-bacterial [27] effects.

Additionally, our study also investigated dose-dependent effects. Drug concentration can profoundly influence its effect on biological responses, and this is an understudied yet critical aspect in CBN pharmacology. To address this gap, we explored the transcriptomic changes induced in neuroblastoma × spinal cord (NSC-34) cells after exposure to different concentrations of CBN compared to controls. NSC-34 is a hybrid cell line generated through the fusion of neuroblastoma cells with motor neuron-enriched embryonic spinal cord cells. It is widely acknowledged both as a model for motor neuron biology and as a useful in vitro system for investigating neurological diseases and injury-related processes [28,29]. Upon differentiation, these cells exhibit morphological and physiological features typical of primary motor neurons [30], making them suitable for exploring neurodegenerative processes. In particular, the analysis focused on biological networks exhibiting CBN-associated dysregulations, with further investigation into their behavior across different dosages. Elucidating these molecular pathways could provide critical insights into the mechanisms underlying CBN’s potential therapeutic effects, paving the way for its application in treating neurodegenerative diseases.

## 2. Materials and Methods

### 2.1. Extraction and Isolation of CBN

*C. sativa* L. was cultivated in a greenhouse at CREA-CIN, Rovigo, IT, and harvested in November 2013. In accordance with its legal status (Authorization SP/106 23/05/2013 from the Ministry of Health, Rome, IT), the cannabinoid was isolated and purified. CBN, with a purity of 94%, was supplied by PlantaChem Srls (Novara, IT).

**Figure 1:**
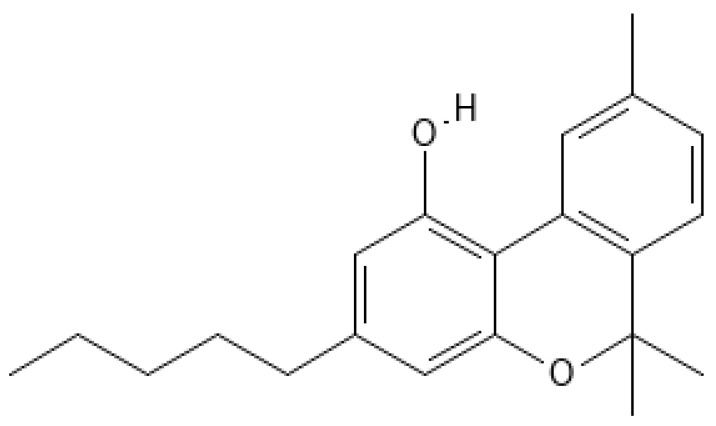
Chemical structure of CBN. The image was created through the use of the PubChem molecule editor tool (https://pubchem.ncbi.nlm.nih.gov/edit3/index.html (accessed on 15 April 2025)).

For this study, **CBN was obtained by chemical synthesis, starting from** non-psychotropic **cannabidiol (CBD)**, following the method described by Pollastro et al. [31]. Briefly, a solution of CBD (100 mg, 0.32 mmol) in toluene (20 mL) was refluxed with iodine (162 mg, 0.64 mmol) for 60 min, with progress monitored by TLC. After cooling, the mixture was washed, and the organic phase was evaporated. CBN (Figure 1) was then purified using silica gel chromatography and HPLC, resulting in 67 mg of CBN with a purity of 99%. The identification of CBN was confirmed through ^1^H NMR (400 MHz, Bruker^®^, CDCl3). For reference, metabolomic profiling from Choi et al. [32] was utilized. All chemicals and solvents were used as received, and purification methods included TLC and HPLC with silica gel columns.

### 2.2. NSC-34 Culture

The NSC-34 was employed in this study to evaluate the effect of the compound in a model reflecting a cellular state closer to the neuronal cell type. NSC-34 is a hybrid cell line obtained by fusing mouse neuroblastoma cells with motor neuron-enriched embryonic spinal cord cells. NSC-34s were obtained from Cellutions Biosystem Inc., Cedarlane (Burlington, ON, Canada) and cultured in high-glucose Dulbecco’s Modified Eagle’s Medium (DMEM) (#D5671, Sigma-Aldrich, St. Louis, MO, USA), supplemented with 10% fetal bovine serum (FBS) (#F7524, Sigma-Aldrich, St. Louis, MO, USA), 1% L-glutamine (#G7513, Sigma-Aldrich, St. Louis, MO, USA) and 1% penicillin/streptomycin (#P0781, Sigma-Aldrich, St. Louis, MO, USA). Cells were maintained at 37 °C in a 5% and CO_2_ 95% air, and were subcultured every 2–3 days upon reaching 80–90% confluency.

### 2.3. Cell Differentiation and Treatment

To better mimic the physiological conditions of mature motor neurons, differentiation was induced using retinoic acid (RA). Cellular differentiation was induced using 1:1 DMEM/F12 (Ham) (#D6421, Sigma-Aldrich, St. Louis, MO, USA), 1% L-glutamine, 1% penicillin/streptomycin, and 1% FBS spiked with 1 µM all-trans RA (atRA) (#R2625, Sigma-Aldrich, St. Louis, MO, USA). The differentiation medium was renewed every 2 days. After 5 days of differentiation, the NSC-34 cell line was treated for 24 h with different concentrations of CBN.

Prior to use, pure CBN was dissolved in dimethyl sulfoxide (DMSO) (#D8418, Sigma-Aldrich, St. Louis, MO, USA) and subsequently diluted with phosphate-buffered saline 1X (PBS) (#806552, Sigma-Aldrich, St. Louis, MO, USA) to achieve an intermediate concentration of 353.97 µM (the final concentration of DMSO was <0.1%). Then, the tested concentrations were diluted in complete 1:1 DMEM/F12 (Ham) without atRA to the final amounts of 5, 10, 20, 50, and 100 µM.

### 2.4. 3-(4,5-Dimethylthiazol-2-yl)-2,5-Diphenyltetrazolium Bromide (MTT) Test for CBN Cytotoxicity and Neuroprotection

The 3-(4,5-dimethylthiazol-2-yl)-2,5-diphenyltetrazolium bromide (MTT) colorimetric assay, based on the reduction in 3-[4,5-dimethylthiazol-2-yl]-2,5-diphenyltetrazolium bromide by oxidoreductase enzymes, was performed to assess the potential effect of CBN on NSC-34 cells. The MTT assay is widely used to assess metabolic activity, providing insights into cell viability, proliferation, and cytotoxicity [33].

NSC-34 cells were seeded in 96-well plates (20 × 10^3^ cells per well) and were differentiated as described below. After differentiation, the cells were treated with different concentration of CBN for 24 h (5, 10, 20, 50, and 100 µM), followed by incubation at 37 °C for 4 h with fresh medium supplemented with 0.5 mg/mL MTT reagent (#M5655, Sigma-Aldrich, St. Louis, MO, USA). Next, the insoluble formazan crystals were dissolved with 0.04 N HCl/isopropanol solution, and the absorbance was measured at 570 nm using a BioTek Synergy H1 microplate reader spectrophotometer (BioSPX, Beersel, Belgium). Eight independent replicates were conducted for each experimental condition. Results are expressed as a relative percentage of cell viability compared to CTRL.

### 2.5. Protein Extraction and Western Blot Analysis

Cells cultured in 6-well plates specifically for Western blot experiments were harvested. After centrifugation, supernatant was discarded, and the pellet was used for protein extraction with RIPA buffer (contents: 25 mM Tris-HCl pH 7.6, 150 mM NaCl, 1% NP-40, 1% sodium deoxycholate, 0.1% SDS) (Thermo Scientific, Waltham, MA, USA) according to the manufacturer’s protocol. Protein extraction was then quantified using the Bio-Rad Protein Assay (Bio-Rad Laboratories, Hercules, CA, USA). After denaturation at 95° C, 20 µg of protein from each sample underwent separation using SDS-polyacrylamide gel electrophoresis (SDS-PAGE). The proteins were then transferred to a PVDF membrane (Immobilon–P, Millipore, Burlington, MA, USA). Membranes were blocked with 5% skim milk in Tris-buffered saline (TBS) for 1 h at room temperature, then incubated overnight at 4 °C with the primary antibody. The antibodies used were as follows: caspase 3 (1:1000; #9662, Cell Signaling Technology, Danvers, MA, USA); cleaved caspase 3 (1:1000; #9664, Cell Signaling Technology, Danvers, MA, USA); anti-active caspase 9 (1:1000; #Ab2324, Abcam, Cambridge, UK); anti-caspase 9 (1:1000; #Ab32539, Abcam, Cambridge, UK); Akt (1:500; #9272, Cell Signaling Technology, Danvers, MA, USA); p-Akt (1:500; #9271, Cell Signaling Technology, Danvers, MA, USA); Nrf-2 (1:500; #sc-722, Santa Cruz Biotechnology, Inc., Dallas, TX, USA); and β III Tubulin (1:1000; #4466, Cell Signaling Technology, Danvers, MA, USA).

Membranes were washed with TBS 1X and incubated with horse radish peroxidase (HRP)-conjugated anti-rabbit IgG secondary antibody (1:2000; #sc-2357, Santa Cruz Biotechnology, Inc., Dallas, TX, USA) or HRP-conjugated chicken anti-mouse IgG secondary antibody (1:1000; #SA1-72021; ThermoFisher Scientific, Rockford, IL, USA) for 1 h at room temperature. To analyze if blots were loaded with equal amounts of proteins, membranes were incubated with HRP-conjugated anti-glyceraldehyde 3-phosphate dehydrogenase (GAPDH), rabbit monoclonal, and 14C10 (1:1000; #3683, Cell Signaling Technology, Danvers, MA, USA). The respective protein bands expression was analyzed through an enhanced chemiluminescence system (Luminata Western HRP Substrates; Millipore, Burlington, MA, USA). Protein bands were acquired using the ChemiDoc™ MP System (Bio-Rad Laboratories, Inc.) and quantified using ImageJ v. 1.54j software.

### 2.6. Total RNA Extraction and cDNA Library Preparation

NSC-34 cells were seeded in 6-well plates at a density of 5 × 10^5^ cells per well. Following differentiation induced by atRA, the cells were treated with CBN, and subsequently, cell pellets were collected using a 0.25% trypsin–ethylenediaminetetraacetic acid (EDTA) solution (#T4049, Sigma Aldrich, Saint Louis, MO, USA). The Maxwell^®^ RSC simply RNA Cells Kit (#AS1390, Promega, Milan, IT) was used to extract RNA from NSC-34 cells, following the manufacturer’s instructions. The cDNA library preparation was performed using the TruSeq RNA Exome protocol (Illumina, San Diego, CA, USA) [34]. RNA samples were subjected to fragmentation for 8 min at 94 °C. First-strand cDNA was synthesized using SuperScript II Reverse Transcriptase (Invitrogen, Milan, IT). Then, the second strand of cDNA was generated and purified using AMPure XP beads (Beckman Coulter, Brea, CA, USA). Subsequently, the 3′ ends of the cDNA were adenylated, and index adapters were ligated to these terminal ends. The AMPure XP beads were used to purify the libraries, and the quality of the cDNA library was validated using the TapeStation 4150 instrument (Agilent, Richardson, TX, USA). The library was then normalized to 1.42 pM. Finally, the samples were sequenced on the Illumina NextSeq 550Dx instrument using the NextSeq 500/550 Mid Output Reagent Kit v2 (300 cycles) in paired-end mode.

### 2.7. MTT and WB Data Analysis

For the MTT test and Western blot, statistical analysis was performed using GraphPad Prism version 6.0 software (GraphPad Software, La Jolla, CA, USA). Statistical analysis was performed using a one-way ANOVA test followed by Bonferroni’s post-hoc test for multiple comparisons. A *p* value of ≤0.05 was considered indicative of statistical significance. Results are presented as mean ± SEM from N-independent experiments.

### 2.8. Transcriptomic Analysis

RNA-seq analysis was performed through a next-generation sequencing Illumina NextSeq550 instrument on cell extracts treated with different CBN concentrations (5, 10, 20, 50, and 100 µM) vs. controls. Data quality was assured by the fastQC v.0.12.0 tool, and following quality filters and probe pruning were performed by Trimmomatic v.0.40 [35]. Spliced Transcripts Alignment to a Reference (STAR) RNA-seq aligner 2.7.10a (New York, NY, USA) [36] was used for read alignment using GRCm39 as the reference mouse genome assembly. Htseq v2.0.5 (European Molecular Biology Laboratory (EMBL), Heidelberg, DE) was used for reads counts [37]. Finally, the DESeq2 v.1.40.2 tool in R was used for differential gene expression analysis [38]. Differentially expressed genes (DEGs) between the two groups were identified based on an adjusted *p* value below 0.05. The Benjamini–Hochberg procedure was applied to correct for multiple testing.

### 2.9. DEGs Filtering

DEGs identified in each comparison (5 µM CBN vs. CTRL, 10 µM CBN vs. CTRL, 20 µM CBN vs. CTRL, 50 µM CBN vs. CTRL, and 100 µM CBN vs. CTRL) were combined and filtered to select genes whose expression is consistently dysregulated across different CBN dosages. Specifically, DEGs obtained from transcriptomic analysis were filtered according to their behavior at multiple dosages. Three factors were considered for the filtering step.

(1) Significance by dosage: This filter retained only DEGs that remained significant at all dosages higher than the first dosage where significance was detected. For example, a gene was retained if it resulted significantly at 20, 50, and 100 µM dosages, whereas a gene that resulted significantly at 10, 50, and 100 µM, but not at 20 µM, was excluded by this step. The rationale behind this step is that if a gene is genuinely influenced by CBN, its dysregulation should persist at all higher concentrations. This filtering step helped remove genes whose expression changes were likely driven by factors unrelated to CBN.
(2) Expression behavior: This criterion allowed for the selection of genes exhibiting different expression patterns at varying dosages. The reasoning behind this is that at very high CBN concentrations, molecular feedback mechanisms may be triggered, altering gene expression patterns compared to lower doses.
(3) Dose–effect relationship: To account for the lax filtering introduced in the second step, we further refined the selection by retaining only genes that showed a consistent increase, decrease, or approximately linear trend across dosages. Genes with highly variable expression patterns (e.g., upregulated at 5 µM, downregulated at 10 µM, upregulated again at 20 µM, etc.) were excluded in this step.

### 2.10. Bioinformatic Analyses

The DEGs that passed the filtering criteria were further analyzed to identify significantly enriched pathways and to determine whether these pathways were inhibited or activated. Over-representation analysis (ORA) was employed to provide a general overview of the significantly enriched pathways according to the DEGs obtained. Then, we used a second approach using signaling pathway impact analysis (SPIA) for each dosage’s data to estimate the direction and the intensity of the perturbation within such pathways in relation to the dose.

The GSEApy package v.1.1.3 [39] in the Python v.3.13.0 environment was used to perform ORA. The graphite package v.1.50.0 [40,41] and the SPIA package v.2.56.0 [42] in the R v.4.5 environment were used to perform SPIA. Pathway information was retrieved using the Reactome 2024 Mouse reference database (https://www.reactome.org/). Pathways were deemed significantly perturbed (either inhibited or activated) if their adjusted *p* value was <0.05. To minimize the likelihood of false positives, *p* values were adjusted using the family-wise error rate (Bonferroni) correction.

Plots were produced in the Python environment, using matplotlib v.3.8.4 [43] and seaborn v.0.13.2 [44] packages.

## 3. Results

### 3.1. Viability Assay Results

**Figure 2:**
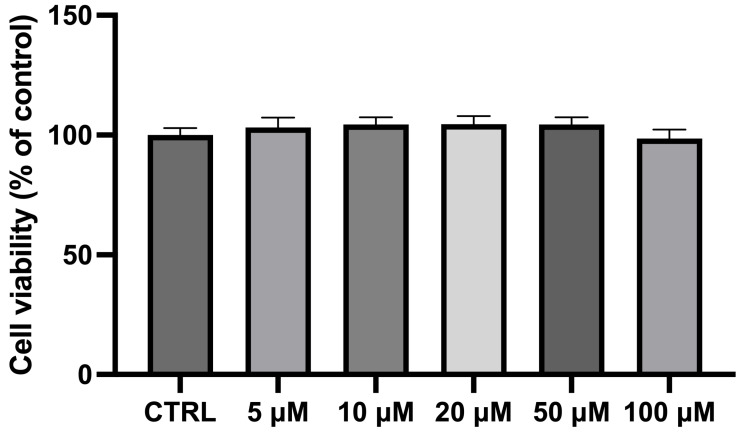
Bar plot of the MTT assay for cell viability after incubating differentiated NSC-34 motor neurons with CBN at different dosages. Cell viability of differentiated NSC-34 motor neurons treated for 24 h with increasing concentrations of CBN (5, 10, 20, 50, and 100 µM) assessed by MTT assay. Spectrophotometric absorbance results are expressed as a percentage of untreated control (CTRL) as the mean ± SEM of eight independent experiments. Results are expressed as percentage of untreated control (CTRL) and shown as mean ± SEM from eight independent experiments.

The exposure of differentiated NSC-34 cells for 24 h to different CBN concentrations (in the range 5–100 µM) did not show any significant variation in cell viability compared to the CTRL cells. As previously observed [45] and reported in Figure 2, CBN did not affect cell viability at 5, 10, 20, 50, and 100 µM concentrations. Notably, these findings are consistent with our previous study on undifferentiated NSC-34 cells [46], further supporting the non-cytotoxic nature of CBN across different experimental conditions. The consistency of these results highlights the reliability of our viability assays and reinforces the idea that CBN may have a favorable safety profile, making it a promising candidate for future therapeutic applications. NSC-34 cells incubated with dimethyl sulfoxide (DMSO < 0.1%) were also analyzed, revealing no cytotoxicity. These findings are consistent with our prior study, which documented normal morphological characteristics and no significant changes in the transcriptomic profiles following DMSO exposure [47].

### 3.2. Transcriptomic Data Overview

**Figure 3:**
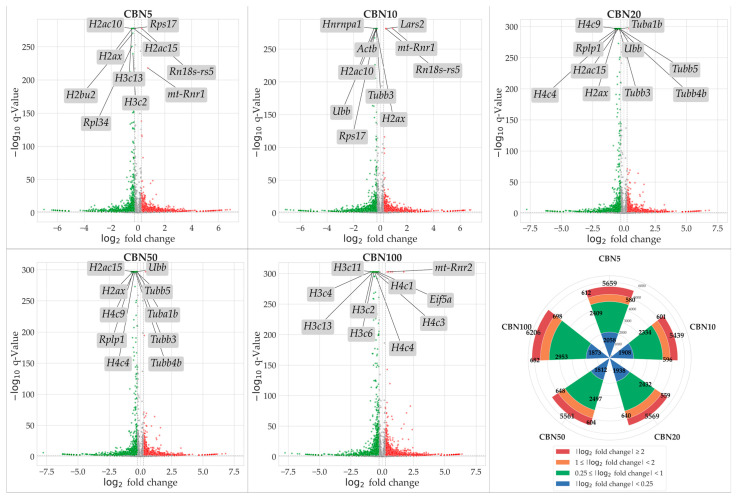
Volcano plots resulted from DEA analyses for each comparison. Upregulated DEGs are shown in red, downregulated DEGs are shown in green; not significantly dysregulated genes are portrayed in grey; and log2 fold change axes are set on −0.25 and +0.25, respectively. The top 10 significant DEGs are also reported in each volcano plot. Additionally, in the figure, we report a radial plot showing the number of significant DEGs for each comparison according to their dysregulation intensity (based on log2 fold change—lFC). Namely, from CBN5 vs. CTRL DEA, we found a total of 5659 DEGs, of which 2058 had a minimal perturbation (lFC < 0.25), 2409 showed low perturbation (0.25 ≤ lFC < 1), 580 showed moderate perturbation (1 ≤ lFC < 2), and 612 showed high perturbation (lFC > 2). From CBN10 vs. CTRL DEA, we found a total of 5439 DEGs, of which 1908 had a minimal perturbation (lFC < 0.25), 2334 showed low perturbation (0.25 ≤ lFC < 1), 596 showed moderate perturbation (1 ≤ lFC < 2), and 601 showed high perturbation (lFC > 2). From CBN20 vs. CTRL DEA, we found a total of 5569 DEGs, of which 1938 had a minimal perturbation (lFC < 0.25), 2432 showed low perturbation (0.25 ≤ lFC < 1), 640 showed moderate perturbation (1 ≤ lFC < 2), and 559 showed high perturbation (lFC > 2). From CBN50 vs. CTRL DEA we found a total of 5561 DEGs, of which 1812 had a minimal perturbation (lFC < 0.25), 2497 showed low perturbation (0.25 ≤ lFC < 1), 648 showed moderate perturbation (1 ≤ lFC < 2), and 604 showed high perturbation (lFC > 2). Finally, from CBN100 vs. CTRL DEA, we found a total of 6206 DEGs, of which 1873 had a minimal perturbation (lFC < 0.25), 2953 showed low perturbation (0.25 ≤ lFC < 1), 698 showed moderate perturbation (1 ≤ lFC < 2), and 682 showed high perturbation (lFC > 2).

To identify genes modulated by CBN treatment, we conducted NGS transcriptomic profiling followed by a differential expression analysis (DEA) for each treatment group (5 µM CBN vs. CTRL, 10 µM CBN vs. CTRL, 20 µM CBN vs. CTRL, 50 µM CBN vs. CTRL, and 100 µM CBN vs. CTRL). Genes exhibiting a statistically significant increase in expression relative to CTRL were considered upregulated, while those showing a significant decrease were classified as downregulated. Statistical significance was adjusted using the Benjamini–Hochberg correction method. A total of 5659 DEGs were observed in the 5 µM CBN vs. CTRL comparison; 5439 DEGs in 10µM CBN vs. CTRL; 5569 DEGs in 20 µM CBN vs. CTRL; 5561 DEGs in 50 µM CBN vs. CTRL; and 6206 DEGs in 100 µM CBN vs. CTRL. Figure 3 reports the results of DEA analyses as volcano plots.

### 3.3. DEGs Selection

This study was structured as a multi-dosage comparison, which permitted us to investigate the behavior of our in vitro models when exposed to different doses of CBN. To better identify genes whose expression is really influenced by this compound, we leveraged the multi-dose data for the DEG selection by giving priority to consistency of dysregulation rather than sheer significance and/or log2 fold changes. Specifically, we applied a filter to select only DEGs showing a consistent dysregulation for each CBN dose (for details, please refer to the Methods section). This filtering step permitted the selection of DEGs exhibiting low but consistent perturbation across conditions, while excluding those with high perturbation observed at only a single concentration. Out of the initial pool of DEGs, 3169 passed this step. These selected genes were subsequently used for the enrichment analyses. Detailed information on the selected DEGs is provided in Supplementary Table S1.

### 3.4. Over-Representation Analysis (ORA)

In this step, the global significance of the DEGs was assessed, regardless of dosage effects, to determine whether they converge on specific pathways. To do so, we used the 3169 DEGs as input for ORA. This analysis focused on the pathways contained in the Reactome reference database for mice. Given the substantial number of significant DEGs, a very conservative approach was adopted to minimize potential false positives and prioritize pathways with a robust association with the experimental outcomes. As such, ORA result *p* values were first corrected with the Benjamini–Hochberg correction; then, we applied a significance threshold of *p* value ≤ ${5\times 10}^{-8}$, a cut-off commonly employed in genome-wide association studies, instead of the normal 0.05 to heighten the stringency of our analysis.

**Figure 4:**
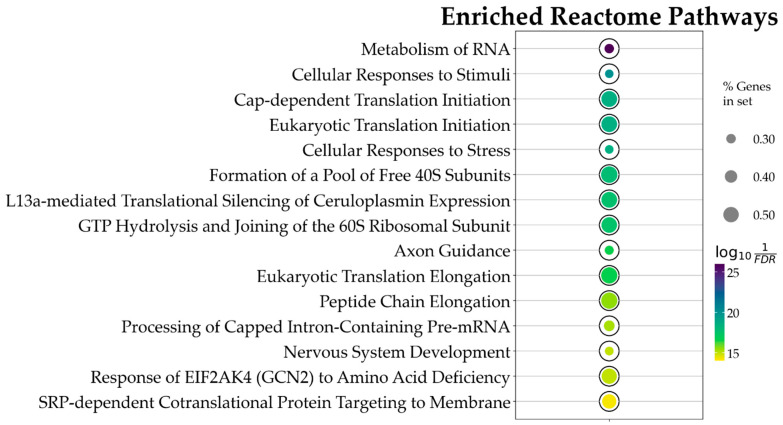
Bubble plot showing the 15 topmost Reactome pathways, ordered by *p* value, associated with CBN action. This result is based on the ORA analysis of the 3169 DEGs. Bubble size represents the ratio of DEGs/total number of genes composing each pathway. Color indicates the significance of the association calculated as ${log}_{10}\frac{1}{FDR}$, where FDR is the *p* value adjusted for false discovery rate with the Benjamini–Hochberg correction method. All pathways here represented have an adjusted *p* value < ${5\times 10}^{-8}$.

Using the above constraints, of the 1862 Reactome pathways, the ORA highlighted 47 significant ones. The data obtained evidence that CBN*’*s main influences in the in vitro model were on transcriptional and translational control, stress response management, and neuronal development and plasticity. Figure 4 and in Table 1 present the top 15 pathways along with detailed results. Due to the relatively large number of identified pathways (47), attention was focused on those most likely involved in neuronal function or dysfunction and on pathways known to be affected by CBN, specifically axon guidance, cellular responses to stress, and cellular responses to stimuli. A comprehensive list of all results from the ORA is provided in Supplementary Table S2.

### 3.5. SPIA Analysis on Selected Pathways (From ORA)

**Figure 5:**
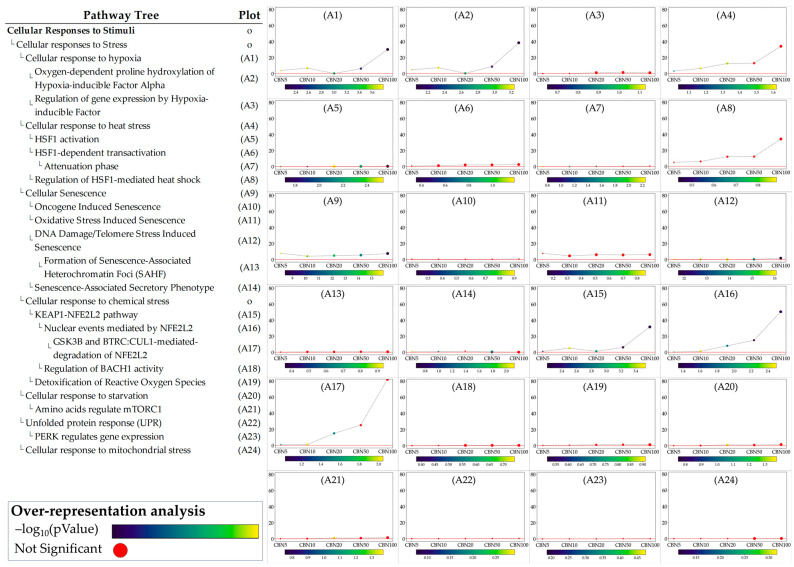
Perturbation plots for the sub-pathways composing the composite process of cellular responses to stimuli. Due to the lack of topological data in the Reactome database, some of the pathways (indicated with “o” in the pathway tree) did not produce any results. On the *y*-axis, we report the perturbation levels. Dot color indicates the over-representation analysis (ORA)-associated *p* value for each dosage. Red dots indicate dosages at which the DEGs were not significantly enriched in the pathway from ORA analysis.

**Figure 6:**
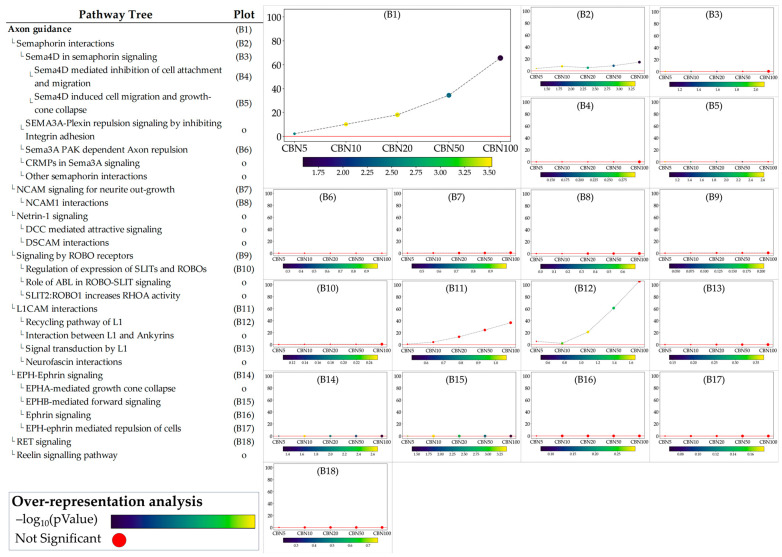
Perturbation plots for the sub-pathways composing the composite process of axon guidance. Due to the lack of topological data from the Reactome database, some of the pathways (indicated with “o” in the pathway tree) did not produce any results. On the *y*-axis, we report the perturbation levels. Dot color indicates the over-representation analysis (ORA)-associated *p* value for each dosage. Red dots indicate dosages at which the DEGs were not significantly enriched in the pathway from ORA analysis.

The ORA step provided general insights into the pathways towards which the selected DEGs converge. While ORA is a useful method to narrow the area of research to the pathways most likely influenced by CBN, it provides limited information on how these pathways are perturbed and how these perturbations vary at the different dosages. To address these aspects, we applied the single pathway impact analysis (SPIA) to the selected pathways. Through this approach, we investigated the level of perturbation of the pathways at the different dosages. It should be noted that SPIA was initially designed for KEGG pathways; while its use on other pathways, even manually set ones, is possible, it is less optimized. This fact in particular affects the direction of the perturbation (intended as activation vs. inhibition). To avoid any bias related to this, we discussed the obtained perturbation scores according to their absolute values, which are sufficient to investigate and evaluate the intensity of the perturbation caused by CBN at various dosages. Furthermore, by leveraging the hierarchical structure of Reactome pathways, we examined the sub-pathways within each selected pathway to determine whether CBN affects the specific clusters of genes. Notably, SPIA analysis leverages pathway topology (nodes and edges). Because some Reactome pathways lack this information, SPIA cannot be applied to them. Figure 5 shows the perturbation plots of the cellular response to stimuli (and stress) pathway tree, while Figure 6 shows those related to the axon guidance. Interestingly, from the data, we observed that the significant signal from cellular responses to stimuli is associated with the perturbation of the cellular response to the hypoxia pathway (A1 in Figure 5) and to the functions related to *Nfe2l2* (Nrf2) (A15–A17 in Figure 5). Likewise, we observed that the entire axon guidance pathway was perturbed (B1 in Figure 6). Within the sub-pathways mostly implicated with axon guidance, the most interesting were related to semaphorins and L1 actions (B2 and B12 in Figure 6, respectively). DEGs were also significantly enriched in the ephrin-related pathways (B14 and B15 in Figure 6); however, the detected level of perturbation was minimal. Supplementary Table S3 reports all the data obtained from SPIA analyses.

### 3.6. Validation by Western Blot

Western blot analysis was conducted on NSC-34 cells treated with increasing concentrations of CBN (5–100 µM) for 24 h, evaluating the protein expression of key markers involved in the oxidative stress response, cell survival, apoptosis, and axon growth indicators.

**Figure 7:**
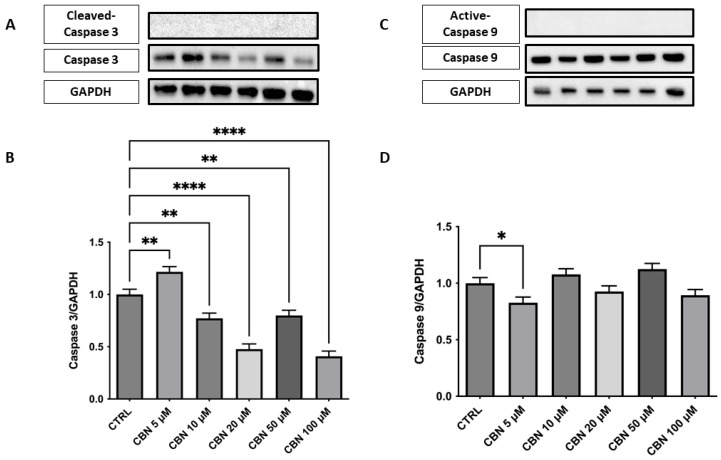
Western blot analysis of caspase-3 and caspase- 9 was performed on NSC-34 cells treated with increasing concentrations of CBN (5–100 µM) for 24 h. (**A**) Representative Western blot images showing protein expression of caspase-3 and GAPDH (loading control). (**B**) Densitometric quantification of caspase-3 expression normalized to GAPDH. A significant decrease in caspase-3 levels was observed at all concentrations of CBN, suggesting reduced apoptotic signaling**. (C)** Representative blots of active caspase-9, total caspase 9, and GAPDH. **(D)** Quantification of caspase-9 normalized to GAPDH showed a modest but significant decrease at 5 µM of CBN. Data are presented as the mean ± SEM from no fewer than three independent experiments. Asterisks (*) indicate *p* value: * *p* < 0.05; ** *p* < 0.01; and **** *p*< 0.0001, respectively.

Analysis of the expression of caspase-3, an important mediator of the apoptotic process, showed a significant dose-dependent reduction in protein levels compared to the control, with the greatest decrease observed at concentrations of 20 and 50 µM (*p* < 0.0001). At 100 µM, a slight increase was observed, while remaining below basal levels. These results are in agreement with the cell viability assays and indicate that CBN does not induce apoptosis at moderate concentrations. This finding could support the hypothesis that CBN, at moderate dosages, may promote cell survival by modulating apoptotic mechanisms (Figure 7A,B). The original membrane images are presented in Supplementary Figure S1. Furthermore, analysis was extended to include the upstream initiator caspase-9, a key regulator of mitochondrial-dependent apoptotic pathways. Western blot analysis showed a mild, statistically significant reduction in active caspase-9 at 5 µM (Figure 7C,D), while higher concentrations did not differ from control levels, suggesting a potential modulation in the intrinsic apoptotic pathway. The original membrane images are included in Supplementary Figure S2. Due to the low signal intensity observed for the cleaved forms of both caspase-3 and caspase-9, Western blot analyses were replicated to ensure the robustness of the validation. A positive control was not available for this set of experiments, and this limitation is acknowledged.

**Figure 8:**
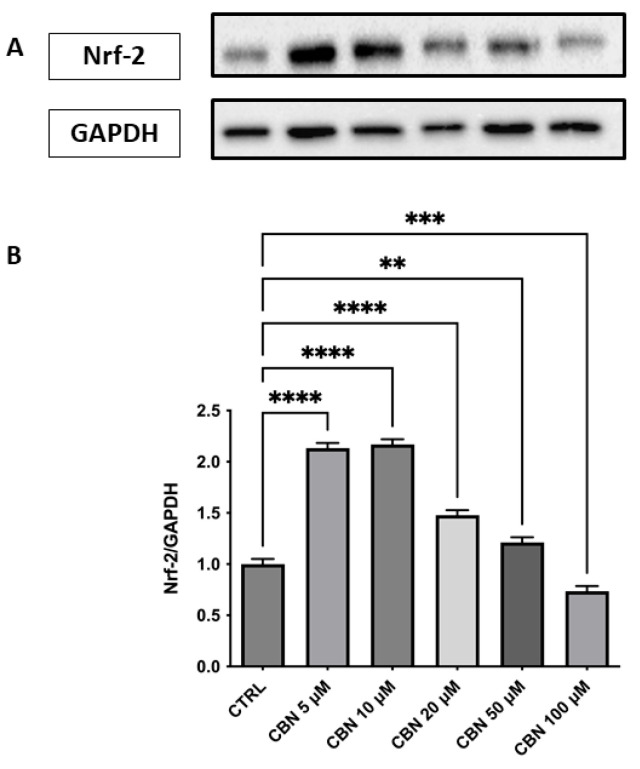
Western blot analysis of Nrf2 protein expression in NSC-34 cells treated with CBN (5–100 µM) for 24 h. (**A**) Representative Western blot bands of Nrf-2 and GAPDH. (**B**) Densitometric quantification of Nrf-2 expression normalized to GAPDH revealed a significant increase at 5, 10, and 20 µM, with the highest expression observed at 10 µM, followed by a decline at 50 and 100 µM., indicating activation of the antioxidant defense response. Data are presented as mean ± SEM from at least three independent experiments. Asterisks (*) indicate *p* value: ** *p* < 0.01; *** *p <* 0.001; **** *p* < 0.0001, respectively.

Regarding the response to oxidative stress, Nrf2 revealed a significant and dose-dependent increase in Nrf2 protein expression, with a peak observed at 5 and 10 µM and a gradual decline at higher concentrations (*p* < 0.0001), as reported in Figure 8A,B. Despite this decrease, Nrf2 levels at 20 and 50 µM remained elevated compared to the control, while at 100 µM, they returned close to baseline. The original membrane images are shown in Supplementary Figure S3. This pattern suggests a transient activation of the Nrf2 pathway, most strongly induced at moderate CBN doses. This finding aligns with transcriptomic data showing an upregulation of *Nfe2l2* gene expression and supports the activation of the Nrf2-dependent antioxidant pathway following CBN exposure.

**Figure 9:**
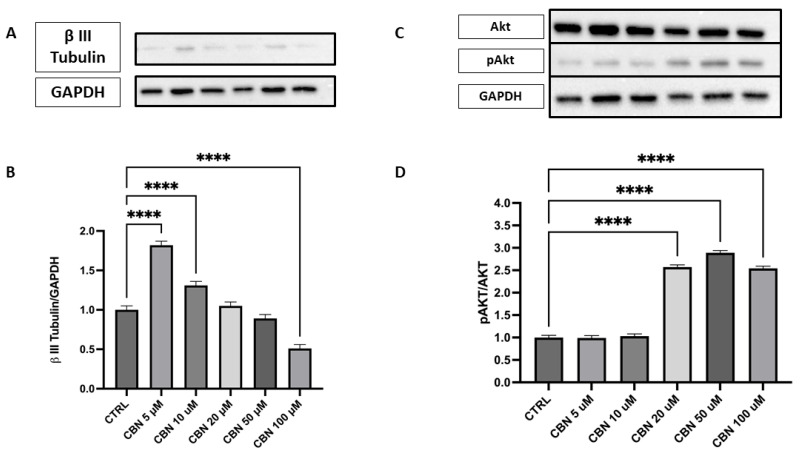
Western blot analysis of β III Tubulin and Akt/p-Akt protein expression in NSC-34 cells treated with CBN (5–100 µM) for 24 h. (**A**) Representative Western blot images showing βIII Tubulin expression and GAPDH (loading control). (**B**) Quantification of β III Tubulin expression normalized to GAPDH. The data indicate a statistically significant increase in β III Tubulin expression following treatment with 5 µM and 10 µM CBN compared to the CTRL (*p* < 0.0001). (**C**) Representative blots of total Akt, phosphorylated Akt (p-Akt), and GAPDH. (**D**) Quantification of the p-Akt/Akt ratio revealed a robust and statistically significant increase at 50 and 100 µM of CBN, indicating activation of pro-survival signaling pathways. However, at higher CBN concentrations, a significant reduction in β III Tubulin expression was observed, with the most pronounced decrease at 100 µM (*p* < 0.0001), suggesting that elevated CBN concentrations may inhibit neuronal differentiation. Data are expressed as mean ± SEM from a minimum of three independent experiments. Asterisks (*) represent levels of statistical significance, with **** corresponding to *p* < 0.0001.

The expression of β III Tubulin protein, a neuronal marker, was assessed by Western blot in cells treated with different concentrations of CBN (5–100 µM) and compared to the CTRL (Figure 9A,B). The data reveal a statistically significant increase in β III Tubulin expression at 5 and 10 µM CBN treatment compared to the CTRL (*p* < 0.0001). However, at high concentrations, a significant decrease in β III Tubulin expression was observed, with the lowest response being seen at 100 µM (*p* < 0.0001). The original membrane images are presented in Supplementary Figure S4. These results suggest that CBN may influence β III Tubulin expression at lower concentrations, but the efficacy seems to decrease at higher concentrations, perhaps due to a saturation effect or other compensatory mechanisms.

Activation of the Akt pathway was investigated via the p-Akt/Akt ratio (Figure 9C,D), a signaling axis crucial for neuronal polarization and dendritic branching. Densitometric analysis showed a robust and dose-dependent increase in the p-Akt/Akt ratio, with the strongest activation at 20, 50, and 100 µM (*p* < 0.0001 compared to control). The original membrane images are shown in Supplementary Figure S5. These results support the involvement of the Akt pathway in CBN-mediated neuroprotection and align with transcriptomic data indicating AKT3 upregulation.

## 4. Discussion

Neurodegenerative disorders currently lack a definitive cure, underscoring the urgent need for novel compounds that can mitigate neurodegeneration, safeguard neuronal cells, and slow or halt progression. In this context, compounds extracted from *C. sativa* garnered the interest of the scientific community due to their potential neuroprotective action. CBN, a minor cannabinoid, exhibits controversial psychotropic effects. While some studies suggest that CBN has minimal psychoactive activity, others indicate that it may produce mild sedative effects. However, research indicates that CBN is approximately 5 to 10 times less potent than THC in terms of psychotropic activity [48,49]. Although cannabinoid receptor activation was not directly assessed, CBN is known to have a higher affinity for CB2 than CB1 receptors, supporting its proposed anti-inflammatory and neuroprotective effects with limited psychoactivity [50,51]. Indeed, chronic inflammation in the brain is deleterious, as it accelerates the progression of several neurodegenerative diseases [52]. However, much of this research is still in the exploratory phase.

Given CBN’s promising neuroprotective potential, the study was designed to investigate its effects on differentiated NSC-34 cells using a well-established in vitro model for evaluating neuronal, particularly motoneuron, responses to therapeutic interventions [29,53]. Additionally, we explored how different concentrations of CBN impact the genes’ expression and the overall transcriptomic profile of these cells, helping us better understand its effects and potential as a therapeutic option. The analysis prioritized the identification of biological pathways exhibiting dose-dependent alterations, as these may elucidate key mechanisms underlying CBN’s effects. Gaining insight into these molecular cascades could open new avenues for the potential therapeutic use of this compound, ultimately enhancing patient health and quality of life.

Following the exposure of differentiated NSC-34 cells to varying concentrations of CBN, cell viability was assessed. The results demonstrate no adverse effects on cellular survival at concentrations up to 100 µM over a 24 h period, including the highest doses tested. The selected concentration range (5–100 µM) was based on previous in vitro studies evaluating cannabinoid activity and is considered pharmacologically relevant without inducing overt CB1 receptor-mediated psychoactivity [45,46]. This suggests its potential safety profile in cellular models, as shown in Figure 2. These findings align with our previous results, which highlight the lack of significant changes in cell viability across all tested concentrations [45].

Indeed, unlike phytocannabinoid derivatives such as CBD or THC, which can induce apoptosis via caspase activation [54,55], CBN did not trigger programmed cell death at any of the concentrations tested. Western blot analysis showed no cleavage of caspase-3 or caspase-9, while total caspase-3 protein levels were markedly decreased (Figure 7A–D), mirroring its transcriptomic downregulation. Caspase-3 is a major effector in the execution phase of apoptosis, whereas caspase-9 is the initiator of the intrinsic (mitochondrial) pathway [56,57]. Their activation leads cells to apoptosis. The absence of any detectable cleaved fragments confirms that CBN does not engage either mitochondrial or executioner caspases up to 100 µM.

The main focus of this investigation was to evaluate the transcriptomic changes related to CBN exposure and identify the biological networks most likely associated with CBN’s action. We performed a multi-dose transcriptomic analysis comparing control and CBN-treated cells at increasing concentrations (5–100 µM).

The results obtained suggest that several pathways (47) may be influenced by CBN administration. Among them, it is worth noting that the pathways of cellular responses to stress, and cellular responses to stimuli and axon guidance are particularly relevant in the context of brain disorders.

According to our data, CBN possesses a pleiotropic effect that ultimately leads to cytoprotective action: CBN was shown to be able to perturb the cellular response to the stimuli pathway. In particular, the genes involved in the response to oxidative stress, such as *Nfe2l2* and *Hmox1* [58]*,* are upregulated by CBN treatment in our model. *Nfe2l2* gene, encoding the Nrf2 protein, is a basic region-leucine zipper transcription factor, initially characterized as the principal regulator of redox homeostasis [59]. The primary regulator of its transcriptional activity is the E3 ligase adapter Kelch-like erythroid cell-derived protein with cap’n’collar homology (ECH)-associated protein 1 (Keap1). Under physiological conditions, Nrf2 levels are kept low because Keap1 binds and inhibits Nrf2 [60]. The Keap1-Nrf2 pathway controls the expression of various protective genes, including those that produce antioxidants, detoxifying agents, proteins that regulate the function of other transcription factors, growth factors, and so forth [61]. This observation was further supported by Western blot analysis, which revealed a dose-dependent increase in Nrf2 protein levels, particularly at 10 µM, peaking at 10 µM and subsequently diminishing at 50 µM, suggesting a complex regulation of Nrf2 at the protein level (Figure 8A, B). The protein-level upregulation of Nrf2 confirms transcriptomic data, reinforcing the role of CBN in enhancing antioxidant defense mechanisms. Notably, due to limitations in the extraction protocols (or similar issues), nuclear and cytoplasmic proteins could not be separated. As a result, it was not possible to determine the concentration of activated Nrf2, which localizes to the nucleus. As such, the WB on Nrf2 should be only intended as validation of the transcriptomic data (which indicated *Nfe2l2* upregulation) rather than a validation of oxidative stress response (for which nuclear Nrf2 would have been needed).

To further confirm the beneficial effects of CBN, we also observed that it downregulates Keap1, the negative regulator of Nrf2, thereby enhancing Nrf2-mediated antioxidant activity.

Nrf2 enhances the expression of antioxidant enzymes, including Hmox1 and Sod, by binding to a specific consensus sequence known as the antioxidant response element (ARE) [62]. Indeed, our transcriptomic data also highlight a dose-dependent upregulation of *Hmox1* and *Sod3* genes. Notably, Sod1, another isoform within the Sod family, was observed to be downregulated following CBN treatment.

The role of Sod1 is not fully understood, but it is hypothesized that enhanced Sod1 activity raises H_2_O_2_ levels, which can become harmful [63]. Additionally, literature observations correlated the upregulation of such isoforms to mitochondrial dysfunction, aberrant RNA metabolism, accumulation of intracellular aggregates, and impairments in axonal transport [64], potentially causing neurological impairments and premature aging [65]. Indeed, in a murine model of ALS, elevated *Sod1* levels were toxic, leading to premature motor neuron death [66,67]. These findings suggest that the double action of CBN in reducing *Sod1* and increasing *Sod3* expressions could prevent intracellular oxidative damage and promote neuroprotective effects.

Notably, the antioxidant and anti-apoptotic effects of CBN aligns are consistent with reports that certain cannabinoids promote neuronal survival, likely through ECS-mediated mechanisms [22,68]. Supporting this, our transcriptomic data suggest that CBN may modulate the axon guidance pathway, which directs axonal growth and ensures proper neural connectivity.

A noteworthy aspect involves the ECS, which has been identified as a key regulator of oxidative stress and balancing apoptotic processes [69]. It is known that the modulation of the ECS could influence the ability of nerve cells to survive or die, and consequently, improve axon guidance. In this way, the ECS may serve as a key regulator of this balance by promoting neuronal cell survival and optimizing axon growth, while simultaneously protecting the nervous system from oxidative stress-induced damage [69,70]. Recent studies further highlighted the role of cannabinoids in axonal growth, showing that cannabinoid receptors and their signaling pathways actively regulate neuronal connectivity during development. [22,70]. Previous studies underscored the role of the endocannabinoid system in axon guidance. Notably, CNR2 has been detected along the retino-thalamic pathway, where it modulates the guidance of retinal axons. The absence of CNR2 in mice results in abnormal retinal projections, highlighting the receptor’s crucial role in the development of retinal connections [71].

In our transcriptomic analysis, we observed that CBN modulates several key regulators of axon guidance, including genes involved in Eph-ephrin signaling and L1-CAM interactions, both critical for neuronal development, synapse formation, and neural regeneration. Specifically, we observed a dose-dependent upregulation of the genes *Vav3, Csnk2a2, Itgb1, Sos1, Col6a2,* and *Grb2* across all tested concentrations. *Vav3*, a member of the VAV gene family, functions as a guanine nucleotide exchange factor for Rho family GTPases, initiating pathways that induce actin cytoskeletal changes and transcriptional modifications [72]. Additionally, *TERF2*, a critical component of the shelterin complex responsible for genomic stability [73], was upregulated following CBN treatment. Interestingly, depletion of *Terf2* could result in the initiation of genomic instability [74]. This suggests that CBN may help maintain chromosomal integrity, potentially preventing telomere dysfunction, a key hallmark of cellular aging and neurodegeneration. Further, although *TERF2* is known for the above functions, recent findings suggest it may also contribute to neuronal differentiation and axonal integrity [75,76]. As demonstrated by the data above, the upregulation of *TERF2* may indicate a neuroprotective effect, enhancing axonal resilience and shielding neurons from DNA damage [76]. Since axon guidance and neuronal survival pathways are closely tied to DNA stability, this suggests a potential protective mechanism mediated by CBN. Further research is needed to determine whether CBN directly regulates TERF2 activity as part of its neuroprotective mechanism. Supporting this hypothesis, our analysis of the axon guidance–semaphorin interaction pathway revealed significant, dose-dependent regulation of multiple genes upon CBN exposure. Semaphorins are extracellular signaling proteins crucial for the development and maintenance of various organs and tissues. Their signaling primarily occurs via plexin receptors, triggering cytoskeletal rearrangements and changes in cell adhesion [77]. Specifically, we observed a significant downregulation of *PlxnA1, Dpysl2, Dpysl3, and Cdk5*, which are involved in axon pruning, growth cone collapse, and neuronal polarity establishment. The reduced expression of these genes suggests that CBN may dampen semaphorin-mediated axonal repulsion, potentially fostering a more favorable environment for axon growth and regeneration. This suggests that CBN modulates the balance between repulsive and attractive cues, thereby promoting neuronal plasticity and synaptic remodeling. Additionally, we observed a downregulation of *RhoB* and *Rnd1*, particularly at higher CBN concentrations (50 and 100 µM). Both genes are critical regulators of axon repulsion and cytoskeletal rearrangement. Given their role in modulating actin dynamics and neuronal polarity, their reduced expression suggests that CBN may influence cytoskeletal remodeling and, consequently, axonal guidance. As shown in the data above, these findings support the hypothesis that CBN actively modulates key axon guidance pathways. By downregulating genes involved in repulsion and cytoskeletal rearrangement, CBN may modify axonal responsiveness to external cues, potentially promoting neurite extension and synaptic plasticity.

In line with this data, Western blot analysis demonstrated that β III Tubulin protein expression was significantly modulated by CBN treatment, showing an increase in its expression at concentrations of 5 µM and 10 µM (Figure 9A,B). This increase suggests that CBN promotes axon stability and growth, a crucial aspect for neuroprotection and neuronal plasticity. β III Tubulin, being a fundamental component of the neuronal cytoskeleton, plays a key role in maintaining axon structure and in their remodeling during synaptic regeneration and plasticity processes [78].

Additionally, we observed that CBN upregulates the Akt3 gene, a key gene involved in various important biological processes, at all concentrations tested in a dose-dependent manner. Notably, Akt is crucial for the regulation of neuronal polarity and branching, as well as metabolism, neurotransmission, synaptic plasticity, and the stress response, including DNA damage repair [79]. Overexpression of Akt in sensory and hippocampal neurons enhances neurite outgrowth and arborization, underscoring its importance in neurodevelopment [80,81]. Western blot analysis demonstrated a significant increase in phosphorylated Akt (p-Akt) levels relative to total Akt upon CBN exposure, with the highest activation observed at 20 and 50 µM, with 100 µM showing a similar but slightly reduced activation (Figure 9C, D).

CBN’s targeted upregulation of both β III Tubulin and Akt, in conjunction with absent apoptotic signaling, suggests a dual role in neuroprotection [82] and in the promotion of axonal growth and neuronal regeneration, as evidenced by the results we obtained on the axon guidance pathway. Ultimately, the interplay between apoptosis, oxidative stress, and axon guidance is critical in understanding the pathophysiology of motoneurons. In this context, oxidative stress induces cellular damage, subsequently triggering apoptotic pathways. This heightened cellular vulnerability promotes motoneuron death, contributing to the progressive loss of motor function. Moreover, oxidative stress has a profound impact on axon guidance, influencing signaling pathways that control the growth and navigation of axons, such as cone dynamics and cytoskeletal rearrangement [83,84].

In this context, our data suggest that CBN may act as a key modulator in the complex interplay between apoptosis, oxidative stress, and axon guidance, with its neuroprotective potential becoming particularly evident at the highest concentrations tested, highlighting its promise as a therapeutic agent in neurodegenerative disorders.

Although CBN is often described as having low psychotropic activity, some studies reported mild THC-like effects at high concentrations. This potential limitation should be considered when evaluating its translational relevance, although the concentrations used in this study fall within a range generally considered sub-psychotropic in vitro. Nevertheless, additional studies are needed to assess whether these effects contribute to functional recovery in neurodegenerative diseases and to clarify the exact molecular mechanisms, indeed, the variability, in CBN’s psychotropic effects that may be influenced by factors such as concentration, individual sensitivity, and the presence of other cannabinoids. Given these uncertainties, it is essential to consider the potential for mild psychoactive effects when evaluating the clinical relevance of CBN-containing products.

## 5. Conclusions

Aging and neurodegenerative diseases are characterized by a progressive decline in cellular functions, including genomic instability, epigenetic alterations, mitochondrial dysfunction, and chronic inflammation. Our study supports that CBN exerts pleiotropic effects by modulating key molecular pathways involved in oxidative stress response, DNA repair, and neuronal survival. These results suggest that CBN positively modulates the response to cellular damage, stimulating the antioxidant response through the Nrf2 pathway and reducing the sensitivity to programmed cell death, as demonstrated by the regulation of caspases and other genes related to neuronal survival. These effects indicate that CBN may be able to support neuronal health under conditions of chronic stress, a hallmark of neurodegenerative diseases. These findings pave the way for further research into CBN’s therapeutic potential, emphasizing the need for in vivo studies to validate its efficacy and safety profile in neurodegenerative disease models.

## Figures and Tables

**Table 1:** Top 15 over-represented pathways selected for further analyses.

Term	Overlap	Adjusted *p* Value	Odds Ratio
Metabolism of RNA	245/761	4.26 × 10^−27^	2.649
**Cellular Responses to Stimuli**	**256/887**	**8.66 × 10^−2^** **^1^**	**2.256**
Cap-dependent Translation Initiation	66/124	3.00 × 10^−19^	6.151
Eukaryotic Translation Initiation	66/124	3.00 × 10^−19^	6.151
**Cellular Responses to Stress**	**229/787**	**3.00 × 10^−^** **^19^**	**2.272**
Formation of a Pool of Free 40S Subunits	59/106	1.56 × 10^−18^	6.775
L13a-mediated Translational Silencing of Ceruloplasmin Expression	62/116	2.31 × 10^−18^	6.200
GTP Hydrolysis and Joining of the 60S Ribosomal Subunit	62/117	3.64 × 10^−18^	6.087
**Axon Guidance**	**169/541**	**2.21 × 10^−^** **^17^**	**2.492**
Eukaryotic Translation Elongation	55/99	2.25 × 10^−17^	6.739
Peptide Chain Elongation	52/94	2.61 × 10^−16^	6.669
Processing of Capped Intron-Containing Pre-mRNA	105/285	5.13 × 10^−16^	3.170
Response of EIF2AK4 (GCN2) to Amino Acid Deficiency	55/106	1.06 × 10^−15^	5.811
Nervous System Development	170/567	1.06 × 10^−15^	2.347

Top 15 pathways enriched from ORA analysis. For each pathway, we report the adjusted *p* value (FDR) and the odds ratio calculated. The database of reference is the Reactome 2024 Mouse database. In bold, we underline the pathways selected for further investigation. The overlap column reports the number of DEGs/the total number of genes within each pathway.

## Data Availability

The data presented in this study can be freely accessed in the NCBI Sequence Read Archive under the BioProject accession number PRJNA1144250.

## Supplementary Materials

The following supporting information can be downloaded at: https://www.mdpi.com/article/10.3390/antiox14060744/s1, Figure S1: Caspase-3 Western Blot; Figure S2: Caspase-9 Western Blot; Figure S3: Nrf-2 Western Blot; Figure S4: β III Tubulin Western Blot; Figure S5: Akt and pAkt Western Blot; Table S1: DEA for CBN Concentration; Table S2: ORA for Reactome pathways; Table S3: SPIA analysis of significant Reactome pathways.
