# Supplementary material for: Cannabinol’s Modulation of Genes Involved in Oxidative Stress Response and Neuronal Plasticity: A Transcriptomic Analysis
Source: Antioxidants (Basel). 2025 Jun 17;14(6):744. doi: 10.3390/antiox14060744 (PMC12189254; doi:10.3390/antiox14060744)
Supplement: Supplementary file 1 [file antioxidants-14-00744-s001.zip › Figure legend.pdf]

Supplementary Figures Legend

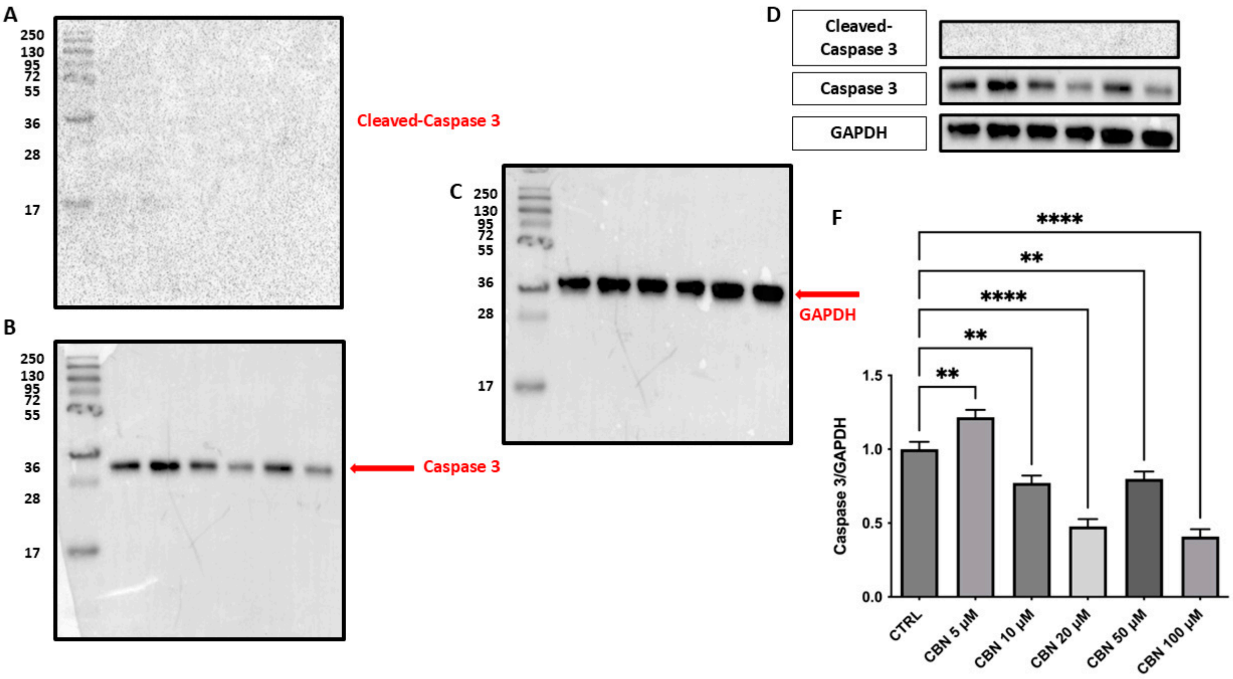

Figure S1. Western blot analysis showing protein expression evaluated on the protein fraction of Caspase 3. On the left-hand side of the figure, the original membrane images are shown for cleaved Caspase 3 (panel A), total Caspase 3 (panel B), and GAPDH. GAPDH, a housekeeping protein, was used to normalize the absorbance values of Caspase 3, ensuring the correction of any technical variations in protein loading. On the right-hand side of the figure, panel D presents representative blots for active Caspase 9, total Caspase 9, and GAPDH. Panel F shows the densitometric analysis of the Caspase 3/GAPDH ratio across different concentrations of CBN treatment, with statistical significance indicated by asterisks (** $p < 0.01$, **** $p < 0.0001$).

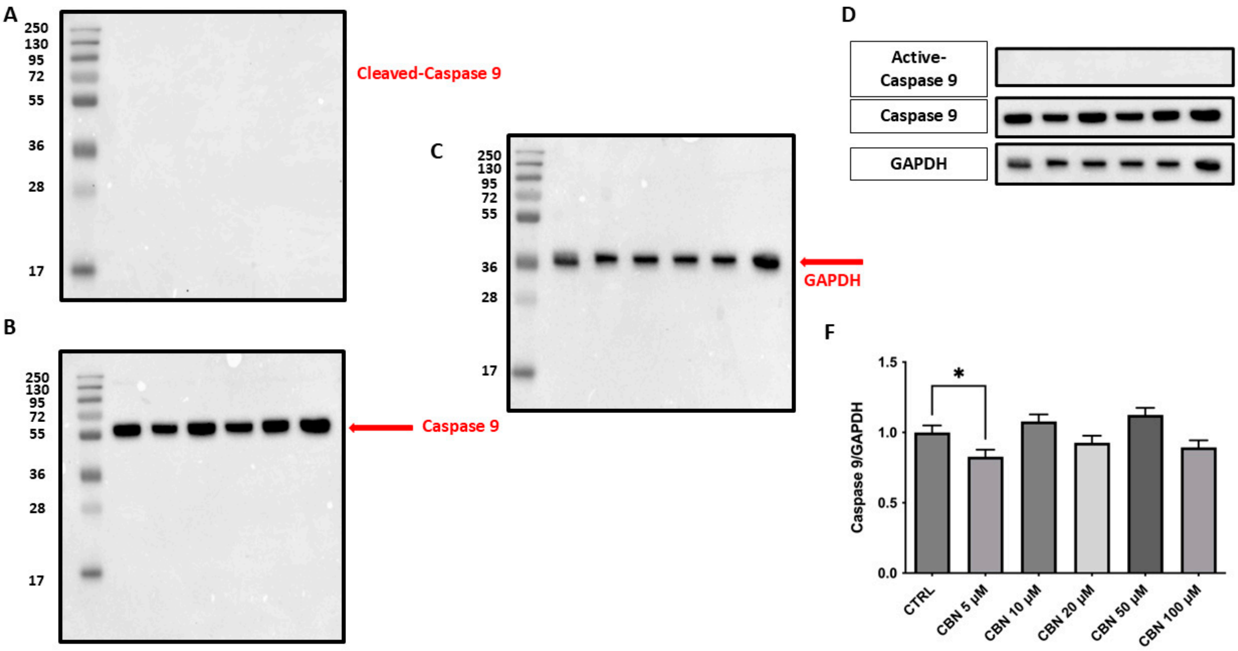

Figure S2. Western blot analysis showing protein expression evaluated on the protein fraction of Caspase 9. On the left-hand side of the figure, the original membrane images are shown for cleaved Caspase 9 (panel A), total Caspase 9 (panel B), and GAPDH. GAPDH, a housekeeping protein, was used to normalize the absorbance values of Caspase 9, correcting for any technical variations in protein loading. On the right-hand side of the figure, panel D presents representative blots for active Caspase 9, total Caspase 9, and GAPDH. Panel F shows the densitometric analysis of the Caspase 9/GAPDH ratio across different concentrations of CBN treatment, with statistical significance indicated by asterisk (*$p < 0.05$).

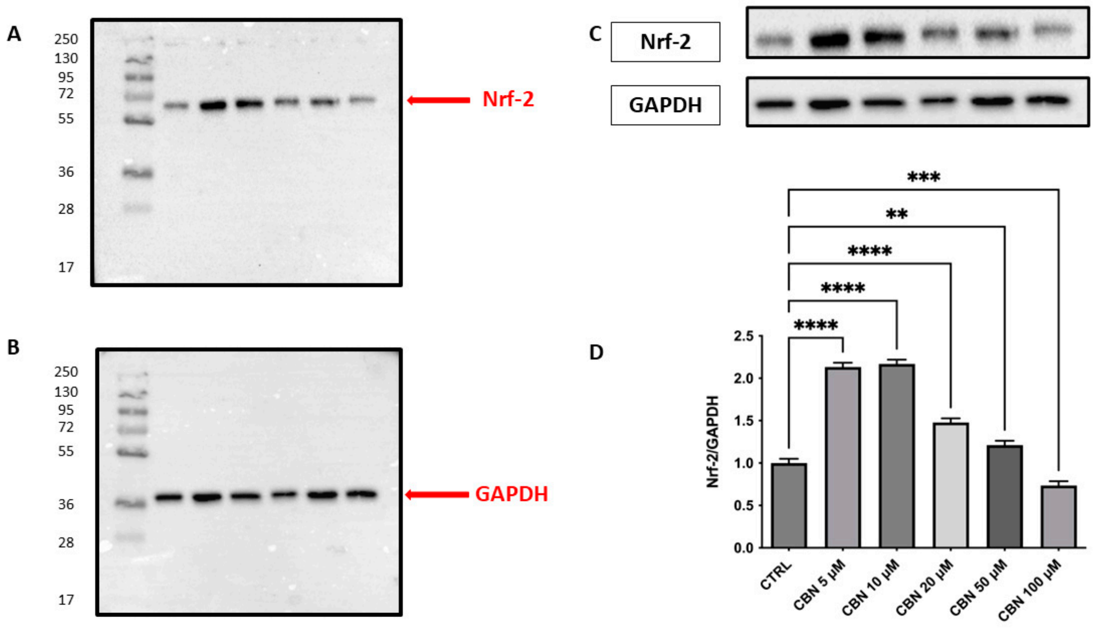

Figure S3. Western blot analysis showing protein expression evaluated on the protein fraction of Nrf-2. On the left-hand side of the figure, the original membrane images are shown for Nrf-2 (panel A) and GAPDH (panel B). GAPDH, a housekeeping protein, was used to normalize the absorbance values of Nrf-2, ensuring the correction of any technical variations in protein loading. On the right-hand side of the figure, panel C presents representative blots for Nrf-2 and GAPDH. Panel D shows the densitometric analysis of the Nrf-2/GAPDH ratio across different concentrations of CBN treatment, with statistical significance indicated by asterisks (**$p < 0.01$, ***$p < 0.001$, ****$p < 0.0001$).

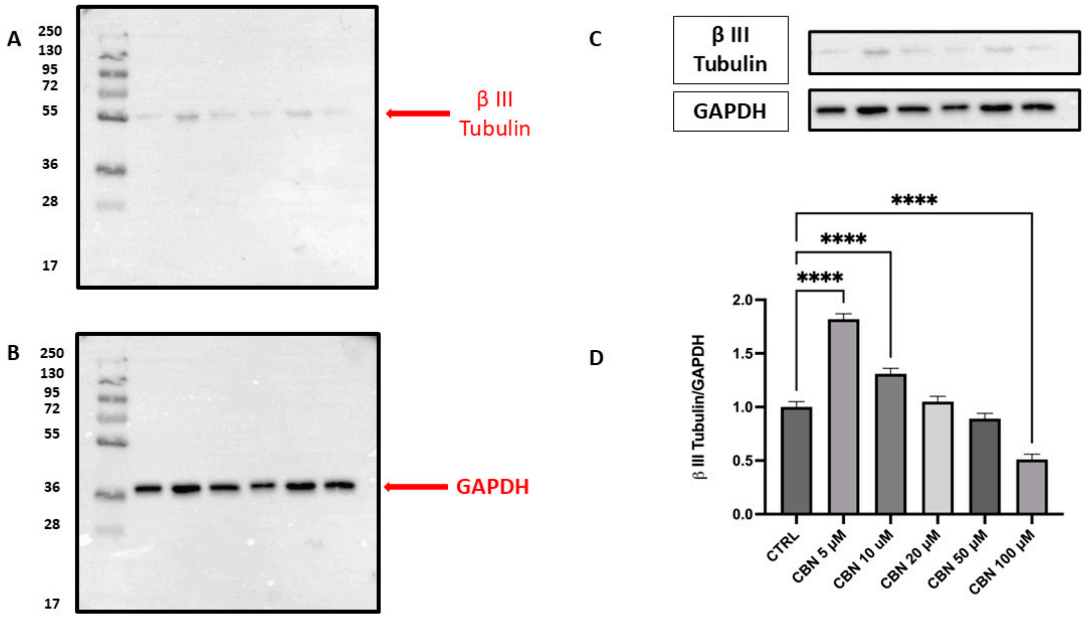

Figure S4. Western blot analysis showing protein expression evaluated on the protein fraction of β III Tubulin. On the left-hand side of the figure, the original membrane images are shown for β III Tubulin (panel A) and GAPDH (panel B). GAPDH, a housekeeping protein, was used to normalize the absorbance values of β III Tubulin, correcting for any technical variations in protein loading. On the right-hand side of the figure, panel C presents representative blots for β III Tubulin and GAPDH. Panel D shows the densitometric analysis of the β III Tubulin/GAPDH ratio across different concentrations of CBN treatment, with statistical significance indicated by asterisks (****$p < 0.0001$).

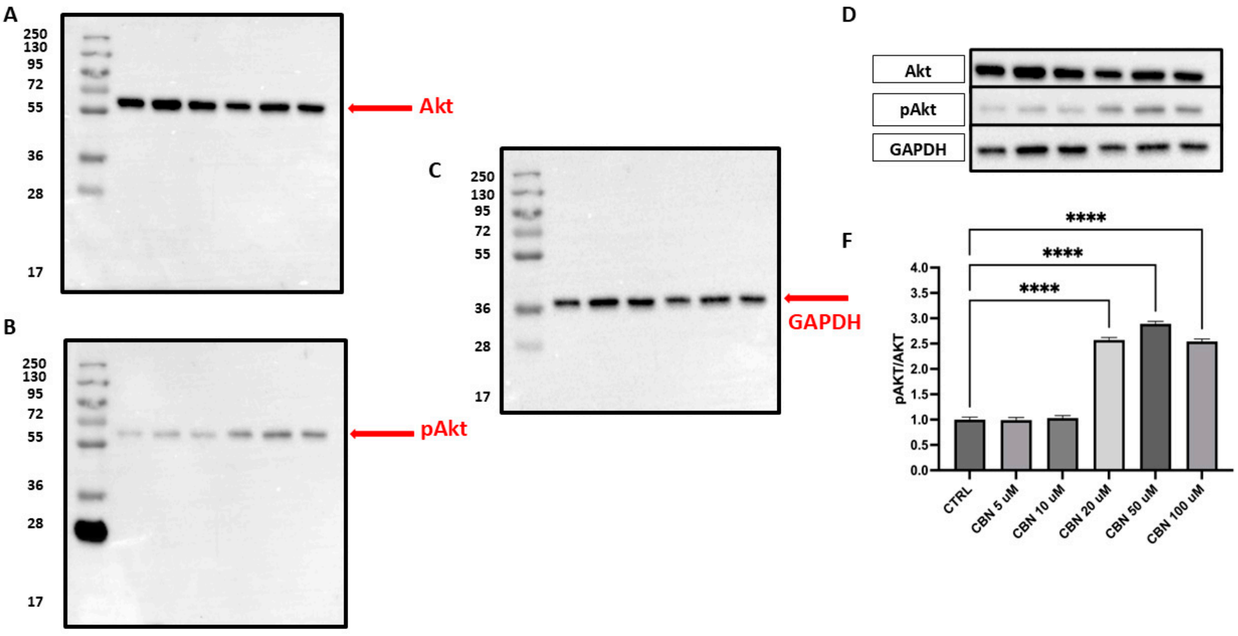

Figure S5. Western blot analysis showing protein expression evaluated on the protein fraction of Akt and pAkt. On the left-hand side of the figure, the original membrane images are shown for Akt (panel A), pAkt (panel B), and GAPDH (panel C). GAPDH, a housekeeping protein, was used to normalize the absorbance values of Akt and pAkt, correcting for any technical variations in protein loading. On the right-hand side of the figure, panel D presents representative blots for Akt, pAkt, and GAPDH. Panel F shows the densitometric analysis of the pAkt/Akt ratio across different concentrations of CBN treatment, with statistical significance indicated by asterisks (****p < 0.0001).
